# A Concept of Thermal Effort for Heat-Induced Metal Plasticity

**DOI:** 10.3390/ma17194824

**Published:** 2024-09-30

**Authors:** Waldemar Dudda, Piotr Józef Ziółkowski, Paweł Ziółkowski, Mateusz Bryk, Janusz Badur

**Affiliations:** 1 Faculty of Technical Sciences, University of Warmia and Mazury, Oczapowskiego 11, 10-719 Olsztyn, Poland; 2 Energy Conversion Department, Institute of Fluid-Flow Machinery Polish Academy of Sciences, Fiszera 14, 80-231 Gdansk, Poland; mbryk@imp.gda.pl (M.B.); jb@imp.gda.pl (J.B.); 3 Faculty of Mechanical Engineering and Ship Technology, Gdansk University of Technology, 11/12 Gabriela Narutowicza Street, 80-233 Gdansk, Poland; pawel.ziolkowski1@pg.edu.pl

**Keywords:** strength differential, stress shearness, equivalent stress, heat-resistive steels

## Abstract

This paper proposes a new concept of material effort that considers heat-induced plasticity for heat-resistant steels. These steels indicate a strength differential effect, a stress shearness effect, pressure sensitivity, and other features. Therefore, a three-parameter, temperature-dependent yield function was presented and, next, analytically and geometrically researched. To validate the accuracy of the formulated yield function, experiments were conducted with the designed specimens to characterize the heat-resistant steels St12T and 26H2MF, which underwent simple shear, uniaxial strain tension, and compression tests. The yield function was calibrated by using a simple analysis. Next, the calibrated constitutive equations were used to numerically determine the load–stroke responses of different tests. The numerical analysis showed that the proposed yield function based on three parameters could accurately describe the thermal effort in various loading conditions from the onset of yielding to the ultimate rupture. Accordingly, the proposed yield function is recommended to model material strength under various thermal loading conditions.

## 1. Introduction

Modern heat-resistant steels are based on a new class of artificial materials and have to fulfill many specific requirements like ultrahigh elasticity, strength, chemical resistivity, heat resistance, life time, and so on [1,2,3]. Therefore, in the literature, many innovative concepts concerning the simultaneous use of rational, experimental, and numerical analysis are often employed for the preparation of advanced design solutions [4]. Heat-resistant steels are typically divided into ferritic, martensitic, and austenitic steels. While ferritic and martensitic steels exhibit a bcc α-lattice structure, austenitic steels feature an fcc γ-lattice structure, which shows a larger number of slip planes and a smaller diffusion coefficient [1,5]. A considerable increase in the operation temperature can be achieved by the application of higher alloyed ferritic–martensitic steels, which feature higher Cr contents exceeding 12%. The application limit of modern ferritic–martensitic 9–12% Cr steels could have been increased to approximately 600 °C for use in turbine applications. For temperatures above 600 °C, only austenitic steels can be applied, since they feature an entirely austenitic lattice structure. These so-called Fe-based superalloys exhibit very beneficial properties, e.g., lower staking fault energy, lower diffusion coefficients and, hence, higher creep resistance, higher Cr and Ni contents for increased oxidation resistance, and improved solid solution hardening compared to ferritic CrMoV steels [6,7,8].

The observed irreversible deformation behaviors of constructive metals usually include strain hardening after the initiation of yielding, including the Baushinger effect, strain rate sensitivity on hardening, thermal softening, damage, and ductile fracture [9,10]. Among them, accurate descriptions of plastic yielding and hardening are of special importance for the accurate modeling of plasticity in various technological processes. However, the deformation characteristics of the structure materials involve complex deformation responses such as the strength differential effect, pressure sensitivity or pressure insensitivity, Lode-parameter dependence, stress triaxiality, and anisotropy in strength and plastic flow [11,12]. Numerous papers have been dedicated to the mathematical modeling of such phenomena—they have been based on complex procedures of model calibration using experiments on specially designed specimens and many paramedical yield functions tested iteratively using numerical calculation and optimization techniques [13,14,15].

The effect of the strength differential is well known in the literature, especially for those materials, such as wood, stones, clays, and marble, which cannot be tensioned too much. In such materials, a state of stress (or strain) describes a material effort that depends on a ratio, $\varkappa =k_{c}/k_{t}$; some limit strengths in compression $\left(k_{c}\right)$ and tension $\left(k_{t}\right)$ probes. Recently, refs. [10,16] observed that, in P91 steel, this effect is considerable (1.18>ϰ>1.20) and should be taken into account even during the designing process for the construction parts of power stations like boilers, pipes, and heat exchangers [17]. It has been found that, at a temperature T = 20 °C, the stress asymmetry is small, while, at 600 °C, it is significantly higher. At both temperatures, the asymmetry increases with an increase in the plastic-equivalent strain ${\bar{\varepsilon }}_{p}$. In failure limits, it has been observed that $k_{c}^{fa}=360;\;k_{t}^{fa}=280$ MPa (ϰ=1.32).

Similarly, the authors of [18], researching the heat-resistive alloy 2CrMoV, found that the asymmetry of the proportionality limit in the laboratory temperature for the elastic compression limit and elastic tension limit $k_{c}^{e}=760$ and $k_{t}^{e}=720$ MPa is equal to $\varkappa =k_{c}^{e}/k_{t}^{e}=1.17$. In a paper [19], the behavior of this ratio was observed for a range of temperatures (20–550 °C). In the papers by Dudda [20,21], the complete data concerning the compression and tension limit behaviors for two important heat-resistive steels, St12T and 26H2MF, were measured; they appeared to have rather unusual temperature-dependent behavior. These are the subjects of the present paper.

The objective of the present paper is to propose a formulation that accounts for thermally induced states of stress in isotropic pressure-sensitive elastoplastic heat-resistive alloys. The present work reviews, compares, and generalizes the past works concerning the so-called energy-based approach, e.g., [22,23,24,25,26,27,28].

In order to enhance the performance of a turbine, the operating temperature needs to be increased. This makes the blade work in difficult conditions. Turbine blades are essential structural parts that form the turbine assembly of a gas or steam turbine engine and help convert the kinetic energy of the steam or burning gases into mechanical energy [29,30]. The efficiency of the turbine is conditioned by the vanes of the turbine blades, and the leading edge of a blade is a site where severe thermal damage is experienced. In operation, the blades are subjected to stresses due to numerous factors, such as very high temperatures of steam or gases, centrifugal force creep, thermal gradients developed due to startup and shutdown, vibration fatigue, and stresses induced due to complexity in geometry and hot corrosion [30,31].

Let us recall that the analysis of thermo-mechanical stresses in turbine blades is a complex task to be investigated. Usually, in the literature, the main attention has been focused on gas turbine blades with large effects caused by varying the cooling air—the flue gas flow that runs through the blade interiors, keeping a constant main stream condition around the blade surface (see [29,32]). Note that rather similar assumptions have been made in the cases of rotors, shafts, or casings (see [18,31,33]). Yet, other simplifications of working conditions should be made in the boilers, where the thermal stresses have a significant impact on efficiency and operating life (see: [34,35,36]).

This paper’s aim is introducing both the strength differential effect and the shear difference (the stress shearness), observed in many heat-resistant steels within the whole range of applied temperatures. Those effects could be described within the frame of the three-parameter ultimate yield surface that strongly depends on temperature. In order to obtain the temperature dependence of three parameters, $k_{t}=k_{t}\left(T\right);\;\varkappa =\varkappa \left(T\right);\;\tilde{\nu }=\tilde{\nu }(T)$, a set of experiments were carried out. The measured value of the plasticity coefficient $\tilde{\nu }(T)$ leads to choosing of a shape of yield surface (elliptical, cylindrical, conical, paraboloidal, or hyperboloidal one) during the process of heating or cooling. However, the measured parameter $\varkappa \left(T\right)$ leads to change in the position of yield surface in the Haigh-Westergaard principal stress space, which means the parameter $\varkappa \left(T\right)$ controls the movement of yield surface along the hydrostatics axis induced by the change in temperature. The tensile ultimate strength $k_{t}\left(T\right)$ describes a decrease in strength with increase in temperature. Those untypical behaviors were systematically researched in this paper.

In Section 2, the revalorized Burzyński model of material effort was analyzed, showing strong temperature dependence of three considered parameters. In particular, the shape, size, and moving position of a yield surface were analyzed. Section 3 is devoted to experimental research; the aim is to determine the characteristic limits $k_{t},\;k_{c},\;k_{s}$ in the function of temperature for two heat-resistive steels. The untypical behavior of two Burzyński parameters ϰ(T) and $\tilde{\nu }(T)$ were visualized and discussed. Section 4 presents temperature-dependent yield surfaces for steels St12T and 26H2MF.

In our experiment, other material data for Chaboche cyclic plasticity were also measured in the function of temperature. In Section 5, the model was firstly validated on experimental data and, next, was used to calculate a response of turbine blade under the action of the thermal cycle. Finally, we have proven that proposed class of yield (paraboloidal movement) surface and plastic potential functions can be introduced into the design process for heat resistive steels as well as the stress shearness and the strength-differential effects.

## 2. A Concept of Thermal Effort

### 2.1. Three-Parametrical Burzyński Hypothesis

Let us introduce the mathematical background usually used in the literature. Reuss introduced a set of tensor invariants in a paper [37]. The main three are $I_{1}=\sigma_{ii}$, $J_{2}=\frac{1}{2}s_{ij}s_{ij}$, and $J_{3}=\frac{1}{3}s_{ij}s_{jk}s_{ji}$ where $s_{ij}=\sigma_{ij}-\frac{1}{3}(\sigma_{kk})\delta_{ij}$. In the literature, different systems of co-ordinates are used based on $I_{1},\;J_{2}$, and $J_{3}$. Most popular is the cylindrical system, ξ, ρ, θ defined as:

(1) $$\xi =\frac{J_{1}}{\sqrt{3}};\;\;\;\rho =\sqrt{2J_{2}};\;\;\;\cos\left(3\theta \right)=\frac{3\sqrt{3}}{2}\frac{J_{3}}{{\left(J_{2}\right)}^{3/2}}\;\;\;\;\;\;\;\;\;for\;0<\theta \leq \frac{\pi }{3},$$

or quasi-Cartesian system, p, q, r:

(2) $$p=-\sigma_{m}=-\frac{1}{3}\;\;\mathrm{tr}\left(\sigma \right)=-\frac{1}{3}\left(\sigma_{1}+\sigma_{2}+\sigma_{3}\right)=-\frac{I_{1}}{3},$$

(3) $$q=\sqrt{2J_{2}}=\sqrt{s_{ij}s_{ji}}=\sqrt{\left[(\sigma_{1}-\sigma_{2}{)}^{2}+(\sigma_{2}-\sigma_{3}{)}^{2}+(\sigma_{3}-\sigma_{1}{)}^{2}\right]},$$

(4) $$r={\left(\frac{9}{2}s_{ij}s_{jk}s_{ki}\right)}^{1/3}={\left(\frac{27}{2}\det s\right)}^{1/3}={\left(\frac{27}{2}\left(\sigma_{1}-\sigma_{m}\right)\left(\sigma_{2}-\sigma_{m}\right)(\sigma_{3}-\sigma_{m})\right)}^{1/3}.$$

Also, octaedric invariants are defined in octaedric co-ordinates as $\sigma_{oct}=\frac{1}{3}\left(\sigma_{1}+\sigma_{2}+\sigma_{2}\right)=\sigma_{m}=\frac{1}{3}I_{1\sigma }$ and $\tau_{oct}=\sqrt{\frac{2}{3}J_{2s}}$. The stress intensity denoted by Novozhilov [38] as $\sigma_{i}$ is equal to $\sigma_{i}=\sqrt{3J_{2}}$, which is identical with the distortional energy equivalent $\sigma_{HMH}=\sqrt{3J_{2}}$ and with von Mises norm $\left|\sigma \right|=\sqrt{3J_{2}}$. It means that $J_{2}=\frac{3}{2}\tau_{oct}^{2}=\frac{1}{2}q^{2}=\frac{1}{3}\sigma_{i}^{2}=\frac{1}{3}\sigma_{HMH}^{2}$, where $\sigma_{HMH}>q>\tau_{oct}$.

The fundamental example, where the invariants appear, is the density of elastic strain energy for so-called Hooke elastic material: $\Phi {=\Phi }_{v}+\Phi_{f}=\frac{1-2\nu }{3E}{\left(I_{1}\right)}^{2}+\frac{1+\nu }{3E}(3J_{2})$. If such energy is a starting point to develop any independent approach, nowadays called an “energy-based” one, one can ask about a physical foundation of this. The rational arguments for defending the generality of energy-based formulations of yield conditions are the following.

The distortional strain energy is usually given by [39]:

(5) $$\begin{array}{c} \Phi_{f}=\frac{1}{12G}\left[{\left(\sigma_{1}-\sigma_{2}\right)}^{2}+{\left(\sigma_{2}-\sigma_{3}\right)}^{2}+{\left(\sigma_{3}-\sigma_{1}\right)}^{2}\right]=\\ =\frac{1}{2G}J_{2s}=\frac{1}{3G}q^{2}=\frac{3}{4G}\tau_{oct}^{2}=\frac{1}{6G}\sigma_{HMH}^{2} \end{array}$$

If the Kirchhoff’s modulus is used as $G=\frac{E}{2(1+\nu )}$, it can be obtained as follows:

(6) $$\Phi_{f}=\frac{1+\nu }{E}J_{2s}=\frac{1+\nu }{3E}\sigma_{HMH}^{2}.$$

and volumetric energy as:

(7) $$\Phi_{v}=\frac{1-2\nu }{3E}(\sigma_{1}+\sigma_{2}+\sigma_{3}{)}^{2}=\frac{1-2\nu }{3E}I_{1}^{2}$$

Yet other formulae are defined in the works by [37,40,41,42].

Note, that energy-based approach was initiated by Beltrami [43] in the form Φ≤K, where Φ is some elastic deformation energy (volumetric density; (J/m^3^)) describing a state of material effort and K is the critical value of this energy. Beltrami firstly found how critical energy K depends on the uniaxial yield $k_{t}$ and torsion $k_{s}$. This approach has also opened the possibility of using many other experimental data like Vickers hardness, Sharpy critical energy (fracture toughness), cohesiveness critical energy, and so on [44].

Next, the concept of “specific work of strain” Φ (internal energy) as a measure of material effort (germ. Die Anstrengung) was developed by Huber in a paper [45], who was able to introduce a notion of “equivalent stress” (or reduced stress $\sigma_{eq}$). Also, he proposed a first picture of a limiting surface within the space of three principal stresses (see: Figure 2 and Figure 1 in Ref. [45]).

It is worth noting that the Helmholtz decomposition $\Phi =\Phi_{v}+\Phi_{f}$ has also established a decomposition of critical energy into ${K=K}_{v}+K_{f}$. Beltrami [43] would have used the word “resilience” to denote the work necessary to be conducted on a body to overcome its elastic forces. The volumetric (cubical) resilience $K_{v}$ is a measure of the work necessary to be expended in compression to increase the density permanently. Distortional resilience $K_{f}$ is the work required to be expended in pure distortion to produce a permanent change of form in the element; it is a limit that $\Phi_{f}$ can reach.

Next, Burzyński [22] has proposed a modification for pressure-sensitive materials where the Poisson coefficient does not appear. Generally, he has proposed a “size function” $\eta_{v}$ correcting the contribution of volumetric energy—contemporary, it is nothing else but introducing the stress triaxiality effect into the energy-based approach. It is one of the main achievements of Burzyński, since he solved a crucial question in a way that does not disturb the scientific power of the energy-based approach [46]. The “size function” $\eta_{v}$ in the hypothesis making the volumetric energy “partially present”; if it is $\eta_{v}=0$, then the material is pressure-insensitive. If $\eta_{v}=1$, then the material is fully pressure-sensitive. Mathematically, the Burzyński hypothesis can be written as:

(8) $$\eta_{v}\Phi_{v}+\Phi_{f}=K$$

where a particular form of pressure dependency of the function $\eta_{v}$ was assumed as:

(9) $$\eta_{v}=\omega +\delta /p$$

and ω, δ are unknown parameters. The core of Burzyński’s idea is to express three unknown parameters ω, δ, K in terms of tripled material limit constants $k_{c},\;k_{t},\;k_{s}$, which are known from the experiment of uniaxial compression, tension, and simple shear. The other forms of the size function could also be considered to find another state of material like brittle and ductile failure or continuous damage. Then, other experimental limits data can be used: bi-axial compression and tension, $k_{cc},\;k_{tt}$; tri-axial compression and tension, $k_{ccc},\;k_{ttt}$; and so on [47].

Recently, Pęcherski and his colleagues proposed extending the Burzyński hypothesis to a hypothesis that takes into account a third invariant, the stress shearness effect, into the energy-based approach and on the Burzyński measure of material effort [13,26]. Trying to find the influence of the Lode parameter, he has proposed introducing a shape function $\eta_{f}$ to make a partial (variable) contribution of the energy density of distortion. It means that the extended material effort hypothesis in the case of variable energy, both with the stress triaxiality effect (volumetric energy) and the stress shearness effect (distortional energy), can be presented as:

(10) $${\overset{ˇ}{\eta }}_{v}\Phi_{v}+{\overset{ˇ}{\eta }}_{f}\Phi_{f}=K$$

where ${\overset{ˇ}{\eta }}_{v},\;{\overset{ˇ}{\eta }}_{f}$ denote the size function and the shape function, respectively. Using the definition of $\Phi_{v},\;\Phi_{f}$ in terms of invariants $I_{1},\;J_{2}$, the condition (Equation (10)) can be expressed in the following way [14]:

(11) $$\eta_{v}\left(I_{1}\right){\left(3I_{1}\right)}^{2}+\eta_{f}\left(J_{3}\right)\sqrt{3}J_{2}=K$$

The shape function (or the Lode influence function) can now be proposed in a mathematical form like P(θ) in the maximum shearness approach, taking, for instance, the two-parameter Podgórski shape function [48]:

(12) $$\eta_{f}\left(J_{3}\right)={\left[\cos\left(\frac{\pi }{6}-\beta \right)\right]}^{-1}\cos\left[\frac{1}{3}\arccos(\alpha \cos\left(3\theta )\right)-\beta \right]$$

and two paramedical size function:

(13) $$\eta_{v}\left(I_{1}\right)=\omega +3\delta /I_{1}$$

Pęcherski et al. [13] have obtained a five parametrical (α, β, ω, δ, K) yield surface (Equation (10)), which is paraboloidal and the cross-section parallel to the octahedral plane indicates that the Lode angle dependence has a hexagonal character. These five unknown parameters are expressed by five experimental data $k_{t},\;k_{c},\;k_{s},\;k_{cc},\;k_{tt}$; the procedure of fitting the paraboloidal yield surface (Equation (11)) has been made using the Levenberg–Marquardt algorithm. Also, the authors of [49,50] have discussed several criteria developed within the energy-based approach in comparison with the Burzyński criterion.

### 2.2. Heat-Induced Yield Surface

Let us start from the Maxwell–Helmholtz decomposition of elastic strain energy in Equations (6) and (7). Using now the Kirchhoff constant, G: 2G(1+ν)=E, $\Phi =\Phi_{v}+\Phi_{f}$, the sum of volumetric and distortional energy, can be written as:

(14) $$\Phi_{v}=\frac{1-2\nu }{12G(1+\nu )}{\left(\sigma_{1}+\sigma_{2}+\sigma_{3}\right)}^{2}+\frac{1}{2}\alpha \Delta T(\sigma_{1}+\sigma_{2}+\sigma_{3})$$

(15) $$\Phi_{f}=\frac{1}{6G}\;\sigma_{HMH}^{2}=\frac{1}{12G}\left[{\left(\sigma_{1}-\sigma_{2}\right)}^{2}+{\left(\sigma_{2}-\sigma_{3}\right)}^{2}+{\left(\sigma_{3}-\sigma_{1}\right)}^{2}\right]$$

where αΔT is the thermal expansion coefficient and increase in temperature. In terms of invariants, $\sigma_{HMH}$ and $3p=\sigma_{1}+\sigma_{2}+\sigma_{3}$ can be written as:

(16) $$\Phi_{f}=\frac{1}{6G}\sigma_{HMH}^{2}=\frac{1+\nu }{3E}\;\sigma_{HMH}^{2}=\frac{1}{2G}J_{2}$$

(17) $$\Phi_{v}=\frac{1-2\nu }{12G(1+\nu )}{\left(3p\right)}^{2}+\frac{1}{2}\alpha \Delta T(3p)$$

In general, the energy-based hypothesis (Equation (17)) is based on three independent limits: $k_{r}$, $k_{c}$, and $k_{s}$. Thus, three unknown parameters ω, δ, K in the Burzyński formula (Equation (8)) can be expressed firstly by $k_{t},\;k_{c},\;k_{s}$ and next by $k_{t},\;k_{c},\;\tilde{\nu }$, where the coefficient:

(18) $$\tilde{\nu }=\frac{k_{r}k_{c}}{2k_{s}^{2}}-1$$

possesses an analogy with the Poisson constant; therefore, it can be called “coefficient of plasticity”. In order to underline that $k_{c}\neq k_{t}$, it can be defined [22] that $\varkappa =k_{c}/k_{r}$, which is called “the strength differential parameter” [51].

Now, let us follow an analytical manner of replacing ω, δ, K with the experimental $k_{t},\;\varkappa ,\;\tilde{\nu }$. Let W be a measure of thermal effort defined as a quasi-linear composition of distortional and volumetric energy: $W\equiv \Phi_{f}+\eta \Phi_{\upsilon }\leq K$, where size function η is dependent on pressure p and two constants ω,δ: η=ω+δ/3p. Using Equations (16) and (17), the following form can be obtained:

(19) $$\begin{array}{c} \frac{1}{6G}\sigma_{HMH}^{2}+\omega \left[\frac{1-2\nu }{12G\left(1+\nu \right)}{\left(3p\right)}^{2}+\frac{1}{2}\alpha \Delta T\left(3p\right)\right]+\\ +\delta \left[\frac{1-2\nu }{12G\left(1+\nu \right)}(3p)+\frac{1}{2}\alpha \Delta T\right]=K. \end{array}$$

Next, the following is introduced:

(20) $$\omega \frac{1-2\nu }{1+v}=\frac{1-2\tilde{\nu }}{1+\tilde{\nu }},$$

and:

(21) $$12GK=\frac{3k_{c}k_{t}}{1+\tilde{\nu }}\;\mathrm{and}\;\delta \frac{1-2\nu }{1+\nu }=\frac{3(k_{c}-k_{t})}{1+\tilde{\nu }}$$

After multiplying Equation (19) by factor $12G(1+\tilde{\nu })$, the following formula can be obtained:

(22) $$\begin{array}{c} \frac{2}{3}\left(1+\tilde{\nu }\right)\sigma_{HMH}^{2}+3\left(1-2\tilde{\nu }\right)p^{2}+3\left(k_{c}-k_{t}\right)+\\ +6\omega G\left(1+\tilde{\nu }\right)\alpha \Delta T\;p+2\delta G\left(1+\tilde{\nu }\right)\alpha \Delta T=k_{c}k_{t} \end{array}$$

Or, shortly:

(23) $$\frac{2}{3}\left(1+\tilde{\nu }\right)\sigma_{HMH}^{2}+3\left(1-2\tilde{\nu }\right)p^{2}+3\left(k_{c}-k_{t}+ak_{r}\right)p+b=k_{c}k_{t}$$

where two parameters a, b are introduced:

(24) $$3ak_{t}p+b\equiv 6\omega G\left(1+\tilde{\nu }\right)\alpha \Delta T\;p+2\delta G\left(1+\tilde{\nu }\right)\alpha \Delta T$$

This means that a, b, introducing the influence of temperature, are defined as:

(25) $$a=2\frac{G}{k_{t}}\omega \left(1+\tilde{\nu }\right)\alpha \Delta T;\;\;\;b=2\delta G\left(1+\tilde{\nu }\right)\alpha \Delta T.$$

Now, in terms of $k_{t},\;\varkappa ,\;\tilde{\nu }$ as well as a, b, yield surface takes a form:

(26) $$f=\frac{2}{3}\left(1+\tilde{\nu }\right)\sigma_{HMH}^{2}+3\left(1-2\tilde{\nu }\right)p^{2}+3k_{t}\left(\varkappa -1+a\right)p+b-k_{c}k_{t}=0.$$

If the invariants $I_{1},\;J_{2}$ are described by the principal stresses, then the expression:

(27) $$\frac{2}{3}\left(1+\tilde{\nu }\right)\sigma_{HMH}^{2}+3\left(1-2\tilde{\nu }\right)p^{2}$$

turns into:

(28) $$\sigma_{1}^{2}+\sigma_{2}^{2}+\sigma_{3}^{3}-2\tilde{\nu }(\sigma_{1}\sigma_{2}+\sigma_{2}\sigma_{3}+\sigma_{3}\sigma_{1}),$$

and a final formula can be written as:

(29) $$\begin{array}{c} \sigma_{1}^{2}+\sigma_{2}^{2}+\sigma_{3}^{3}-2\tilde{\nu }\left(\sigma_{1}\sigma_{2}+\sigma_{2}\sigma_{3}+\sigma_{3}\sigma_{1}\right)+k_{t}\left(\varkappa -1+a\right)\left(\sigma_{1}+\sigma_{2}+\sigma_{3}\right)+b=\\ =k_{c}k_{t} \end{array}$$

The formula above is based on five measured experimental parameters $k_{t},\;\varkappa ,\;\tilde{\nu },\;a,\;b$. The values and ranges of the above parameters were obtained from experimental data. It follows from these that the contribution of a, b parameters are negligible and can be taken to be a, b→0. But, in general, calibration of a, b as a thermally sensitive material is a question like the calibration of models with plastic dilatancy. Remembering that the strength difference ratio is $\varkappa =k_{c}/k_{t}$, the three-parameter hypothesis (26) can be written as:

(30) $$f=\frac{2}{3}\left(1+\tilde{\nu }\right)\sigma_{HMH}^{2}+3\left(1-2\tilde{\nu }\right)p^{2}+3(\varkappa -1)k_{t}\;p-{\varkappa k}_{t}^{2}=0$$

For certain values of $\varkappa ,\;\tilde{\nu }$, Equation (30) describes different types of limit surfaces in the principal stresses space. This directly gives the Burzyński equivalent stress $\sigma_{Bu}$ as a function of $3J_{2}=\sigma_{HMH}^{2}$ and $3I_{1}=p$:

(31) $$\sigma_{Bu}=\frac{1}{2\varkappa }\left\{3\left(\varkappa -1\right)p+\sqrt{9(\varkappa -1{)}^{2}p^{2}+4\varkappa \left[\frac{2(1+\tilde{v})}{3}\sigma_{HMH}^{2}+3(1-2\tilde{\nu })p^{2}\right]}\right\}$$

### 2.3. Parametrical Analysis of Three-Parametrical Yield Surface

**Figure 1:**
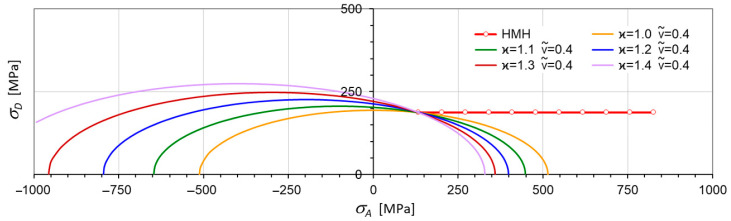
Axiatoric cross-section for $\tilde{\nu }=\mathrm{const}$ (κ variable).

**Figure 2:**
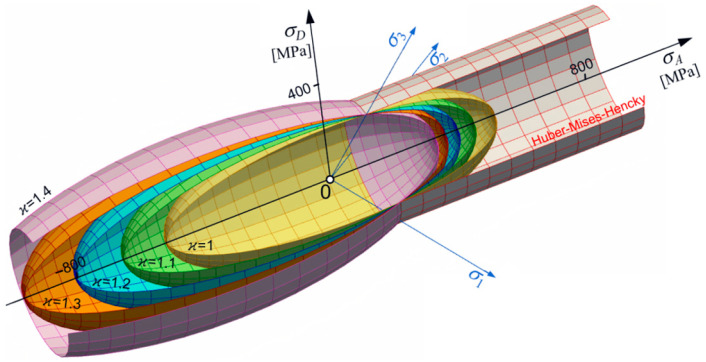
Ellipsoidal surfaces set for $k_{t}=230\;MPa$ and $\tilde{\nu }=0.4$.

Let us consider changes in shape, size, and position of the limit surface (Equation (30)) in the principal space. From simple inspection, it follows that most important is the dimensionless pure shear $k_{s}$ limit defined by $\tilde{\nu }$ (Equation (18)). For $\tilde{\nu }<\frac{1}{2}$, an ellipsoidal surface occurs with one of its axes parallel to the p-axes. For ϰ≠1, the ellipsoidal surface is translated along the p-axes (Figure 1 and Figure 2). For ϰ≥ 1, the strength differential effect indicates that compression is less destructive and the ellipsoidal surface moves proportionally back. For ϰ→∞, the tension domain becomes equal to zero. For $\tilde{\nu }=0.5$, the ellipsoidal surface changes into the Huber–Mises–Hencky infinite cylinder, which, in the axiatoric section, is represented by two straight lines.

**Figure 3:**
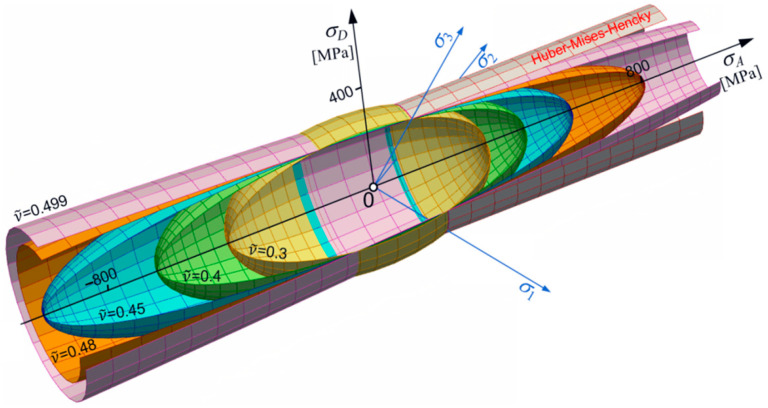
The set of ellipsoids for $k_{t}=230\;MPa$ and ϰ=1.1 ($\tilde{\nu }$ variable).

However, fixed constant strength difference ratio ϰ=const can show the role of $k_{s}$ in creating the shape of the limit surface. For instance, in the elliptical case, the influence of $\tilde{\nu }$ is shown in Figure 3.

Next, if:

(32) $$k_{s}=\sqrt{\frac{k_{t}k_{c}}{3}}$$

then $\tilde{\nu }=1/2$, and a paraboloidal surface can be obtained with an axis parallel to the hydrostatic axis that can translate along it. When, additionally $k_{c}=k_{t}$ and $k_{s}=k_{t}/\sqrt{3}$ ($\kappa =1;\;\tilde{\nu }=1/2$), then Equation (30) turns into the HMH hypothesis $f=\sigma_{HMH}^{2}-k_{t}^{2}=0$ and the yield surface becomes the infinte cirular cylinder. Additionaly, if:

(33) $$k_{s}=\frac{2}{\sqrt{3}}\frac{k_{c}k_{t}}{k_{c}+k_{t}}$$

then the Drucker–Prager cone is obtained [52]:

(34) $$\sqrt{3J_{2}}+\frac{k_{c}-k_{t}}{k_{c}+k_{t}}I_{1}-2\frac{k_{c}k_{t}}{k_{c}+k_{t}}=0.$$

**Figure 4:**
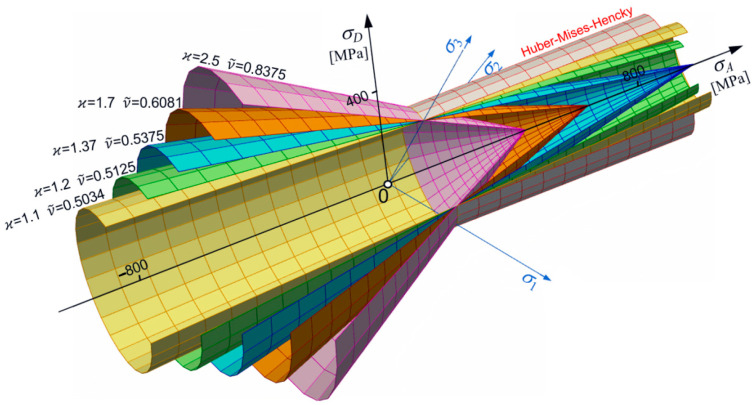
Set of conical surfaces for $k_{t}=230\;MPa$.

The set of conical surfaces is shown in Figure 4. Finally, when:

(35) $$\frac{1}{2}<\tilde{\nu }<\frac{3(k_{c}+k_{t})}{8k_{c}k_{t}}-1$$

the condition (Equation (14)) leads to the hyperboloidal limit surface (one or two sheets) with a physical meaning only in special cases.

**Figure 5:**
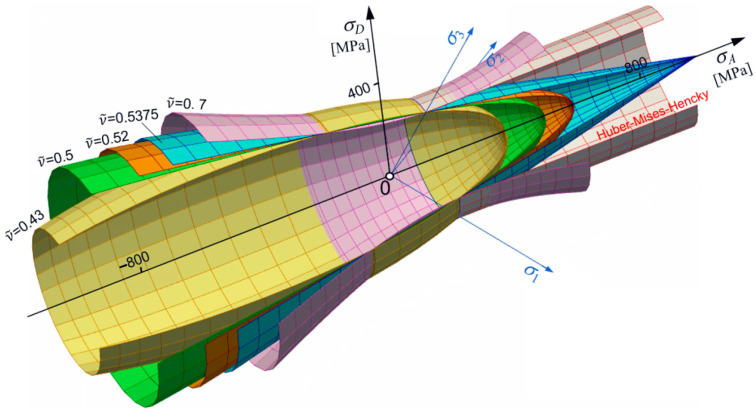
Common presentation of different shapes of limit surface (for $k_{t}=230\;MPa$ and ϰ=1.37, variable $\tilde{\nu }$).

Figure 5 shows the composition of cases for the whole range of changes in coefficient of plasticity for $\tilde{\nu }$. Since $\tilde{\nu }$ changes during thermal load, it means that the shape of the limit surface is not fixed and, in general, can change shape from ellipsoidal to hyperboloidal.

**Figure 6:**
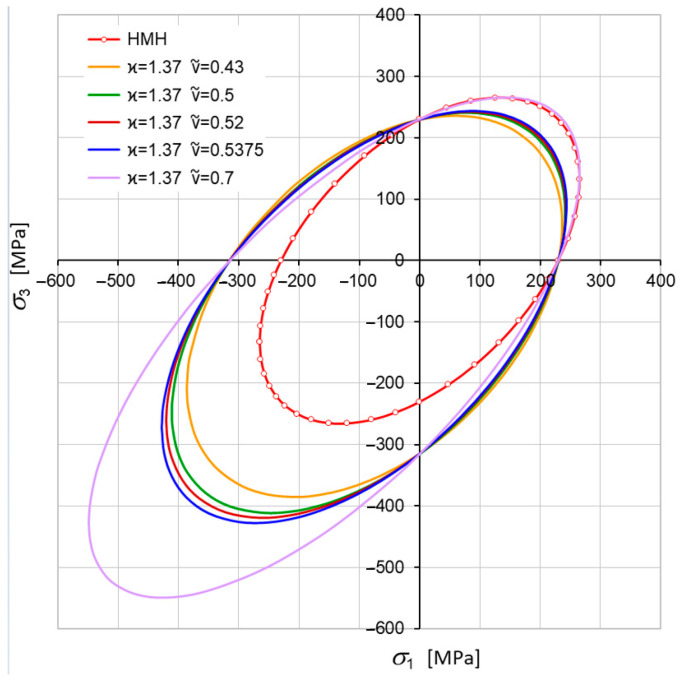
Cross-sections by the plane $\sigma_{2}=0$ for $k_{t}=230\;\mathrm{MPa}$.

Figure 6 presents a cross-section of the plane $\sigma_{2}=0$. Since $k_{t}=const$, then intersections of every curve on the principal axes $(\sigma_{1}$, $\sigma_{3})$ are at the same points $\sigma_{1}=k_{t}$ and $\sigma_{3}=k_{t}$, respectively.

The above analysis shows that the problem of precise experimental determination of pure shear yield $k_{s}$ is crucial, and the value of $k_{s}$ cannot be taken from approximate theoretical formulas any more. For instance, de Saint Venant rule, $k_{s}=k_{t}{\left(1+\nu \right)}^{-1}$, or Beltrami rule, $k_{s}=k_{t}{\left[2\left(1+\nu \right)\right]}^{-2}$ (where ν is the Poisson ratio), cannot be accepted without experimental verification [21]. Also, for materials exhibiting strength difference, any averaging (harmonic: $k_{s}=\frac{k_{r}+k_{c}}{2\sqrt{3}}$; geometric: $k_{s}=\sqrt{\frac{k_{t}k_{c}}{3}}$; arithmetic: $k_{s}=\frac{2}{\sqrt{3}}\frac{k_{t}k_{c}}{k_{t}+k_{c}}$) cannot be taken without verification; this is because small differences in value (about 0.25 MPa) can change the shape of the surface from ellipsoidal to conical.

It is known in the literature that the precision of determination of $k_{s}$ is much lower than the precision of measuring $k_{c}$ and $k_{t}$. Therefore, for some materials, it is easier to determine another limit, for instance, a biaxial compression $k_{cc}$. Then, starting once again from the very beginning, $W\equiv \Phi_{f}+(\omega +\delta /3p)\Phi_{\upsilon }\leq K$ [21], the analytical expression of unknown parameters ω, δ, K can be found in terms of $k_{t},\;k_{c}$, and $k_{cc}$ as:

(36) $$\omega =-\frac{1}{3}\frac{c}{d}\frac{1}{\Delta }(2k_{c}k_{cc}+k_{t}k_{c}-2k_{t}k_{cc}-k_{cc}^{2})$$

(37) $$\delta =-\frac{c}{d}\frac{1}{\Delta }k_{cc}^{2}(k_{c}-k_{t})$$

(38) $$K=\frac{c}{d}\frac{1}{\Delta }k_{cc}^{2}(k_{c}+k_{t})$$

where $c=(1+\nu )E^{-1}$, $d=(1-2\nu ){\left(6E\right)}^{-1}$; $\Delta =2k_{c}k_{cc}+k_{t}k_{c}-4k_{cc}^{2}-2k_{t}k_{cc}$. Knowing the relationship $k_{cc}\sim 2k_{c}$, the above Figure 1, Figure 2, Figure 3, Figure 4, Figure 5 and Figure 6 can be repeated. The above analytical formula can be easily repeated also for other data as $k_{t},\;k_{c}$, and $k_{tt}$. In general, when the number of unknown parameters is greater or equal to five for their identification, it is required to use some optimization methods like the Levenberg–Marqurdt algorithm [13,14,25] or inverse engineering approach [11,12].

## 3. Experiments for Heat-Resistant Steels St12T and 26H2MF

### 3.1. Experimental Expressions for St12T and 26H2MF Heat-Resistant Steels

Uniaxial tension and compression as well as pure-shear (torsion) tests were performed on smooth specimens within five temperatures (20, 200, 400, 600, and 800 °C) to measure the temperature dependence of three parameters of the proposed model: $k_{t},\;\varkappa ,\;\tilde{\nu }$. Two heat-resistant steels, St12T and 26H2MF, have been measured at the original experimental stand. The details of the design, construction, and yield behavior have been described in [21]. Testing conditions used for the loading smooth tests were the same for each temperature investigated.

In Table 1, the chemical composition of analyzed steels is presented.

### 3.2. Uniaxial Tension Test

**Figure 7:**
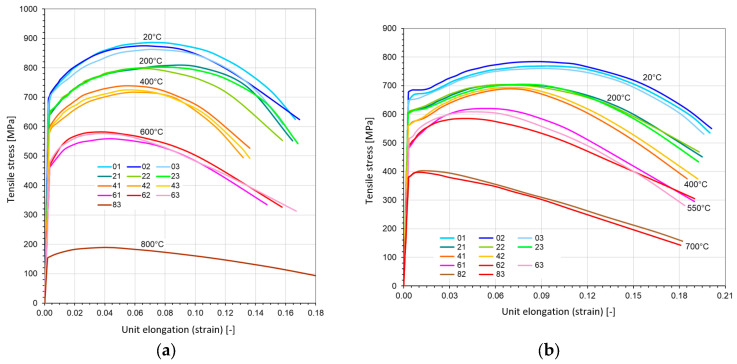
Tension–strain diagrams for three probes in four elevated temperatures: (**a**) made with steel St12T; (**b**) made with steel 26H2MF.

The experimental data of the slow loading given by the true stress–logarithmic strain relationship were measured and calculated. In Figure 7, the experimental curves are pictured for both steels. The changes in the loading slope quantified from the strain $\varepsilon_{0.2}$ were used to determine $k_{t}$ [21]. Strain in those figures is determined as pure elongation: $\varepsilon =\Delta l/l_{0}$.

The exact loading speed was equal to 0.5 mm per minute for tensile test.

### 3.3. Uniaxial Compression Test

**Figure 8:**
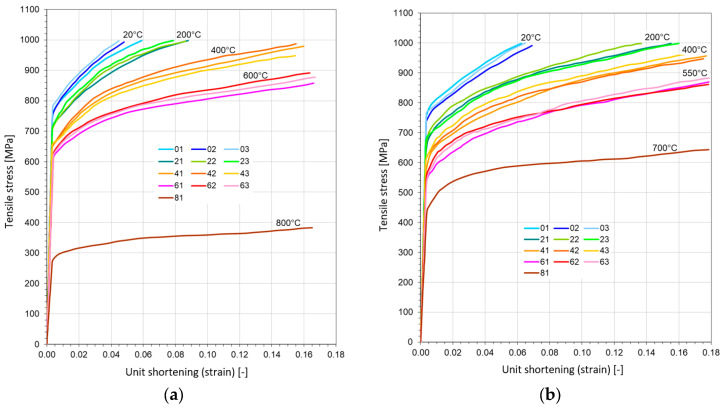
Compression–strain diagrams for three probes in four elevated temperatures: (**a**) made with steel St12T; (**b**) made with steel 26H2MF.

The compression test for both heat-resistive steels was performed in a similar way. Those data were the base for calculating $k_{c}=k_{c}(T)$ curve. In Figure 8, the experimental curves are pictured for both steels.

The exact loading speed was equal to 0.5 mm per minute for compression tests.

### 3.4. Pure Torsion Test

**Figure 9:**
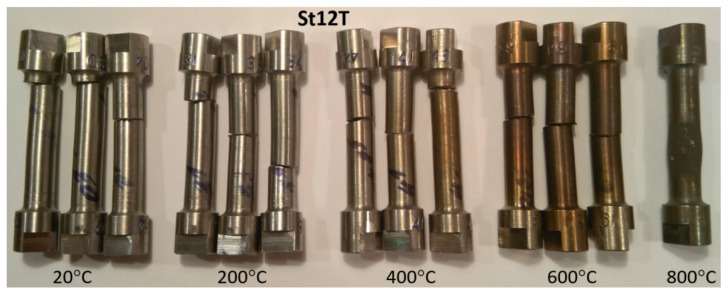
Failure probes of St12T after pure torsion tests.

A special stand was prepared for pure torsion test [21]. Quite analogical, the elastic limits $k_{s}=k_{s}(T)$ have been calculated. Damaged probes after pure torsion tests for St12T steel are presented in Figure 9.

**Figure 10:**
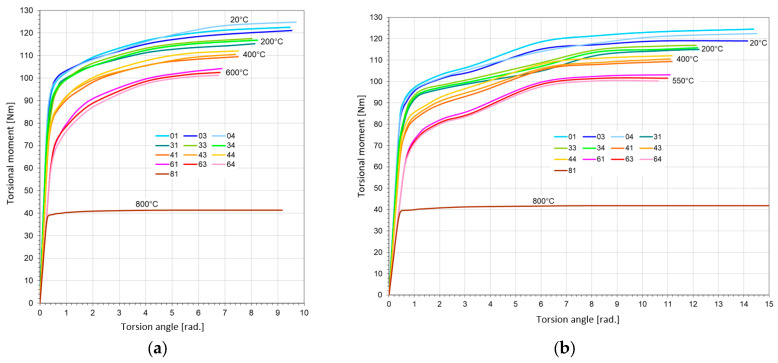
Diagrams of pure torsion tests for probes at elevated temperatures: (**a**) made with steel St12T; (**b**) made with steel 26H2MF.

The elastic limits for pure torsion were calculated from the ${k_{s}=R}_{0.3s}^{T}=M_{0.3s}^{T}/W_{0}$, where $M_{0.3s}^{T}$ is the value of torsion moment for angle γ = $\gamma_{0.3}$ = 0.003 (rad). The averaged values were calculated from three probes—for steel St12T (Figure 10a) and for steel 26H2MF (Figure 10b).

**Figure 11:**
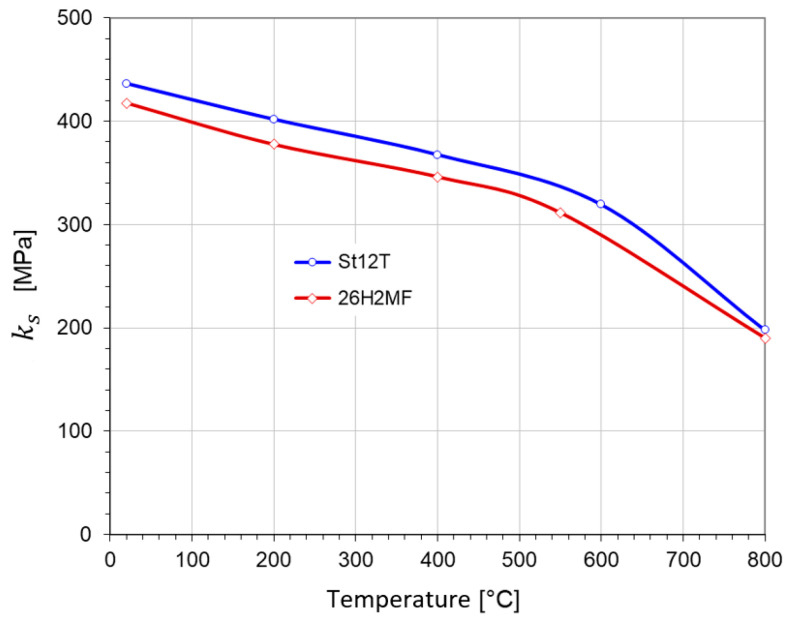
The temperature dependence of plastic limits $k_{s}$ for steels St12T and 26H2MF.

Figure 11 presents the function $k_{s}=k_{s}\left(T\right)$, being the averaged change in elastic limits of pure torsion with temperature calculated for both steels under consideration.

In the pure torsion test, the exact speed loading did not exceed 0.3 rad/min.

### 3.5. Determination of $\kappa (T)\equiv \varkappa_{T}$ and $\tilde{\nu }(T)\equiv \nu_{T}$ Parameters in the Temperature Function

**Figure 12:**
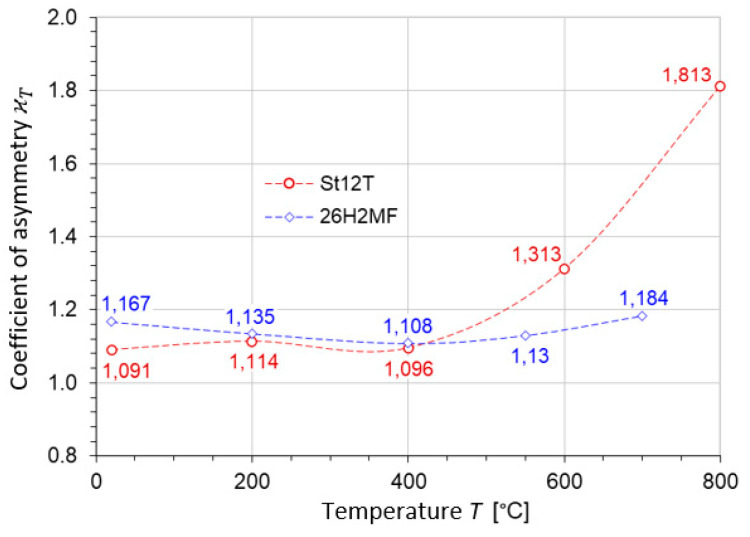
Coefficient of asymmetry for steel St12T and 26H2MF in function of temperature.

**Figure 13:**
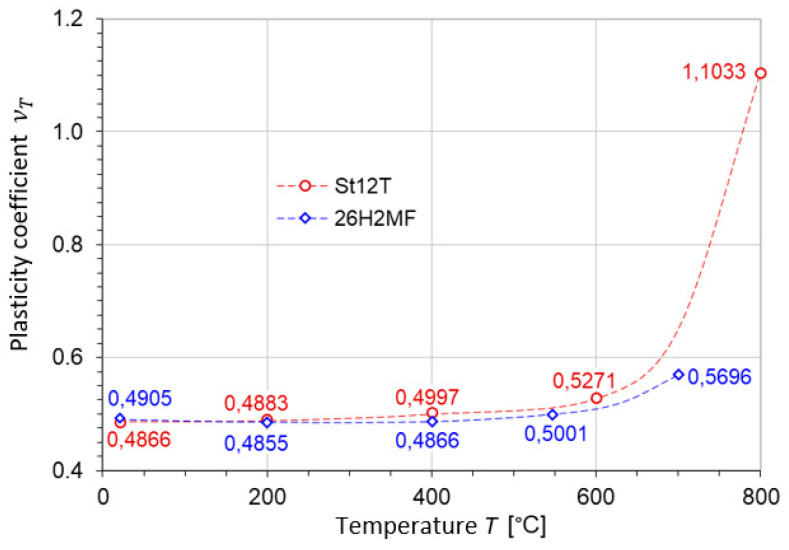
Plasticity coefficient for steels St12T and 26H2MF at elevated temperatures.

The asymmetry coefficient $\kappa_{T}$ and the plasticity coefficient $\nu_{T}$ were determined according to Formula (18) from the measured values of $k_{t},\;k_{c},\;k_{s}$ (see Figure 9, Figure 10 and Figure 11). In Table 2, the plasticity coefficient and the coefficient of asymmetry at elevated temperatures for both steels, St12T and 26H2MF, are presented. The coefficient of asymmetry for steel St12T and 26H2MF is shown as a function of temperature in Figure 12. The plasticity coefficient for steels St12T and 26H2MF at elevated temperatures is presented as a function of temperature in Figure 13.

The experimental data were next used for analysis of the influence of particular parameters on the geometry of the limit surface determined by Equation (30). It is a subject of Section 4.

It is worth mentioning that, for the considered steel St12T at 800 °C, the ductility increases sharply, which is the opposite of what happened up to 600 °C, with a simultaneous large decrease in strength, which is responsible for the sharp increase in the coefficient of asymmetry in Figure 12 and the plasticity coefficient in Figure 13.

## 4. Behavior of Three-Parametrical Yield Surfaces for Experimental Data

**Figure 14:**
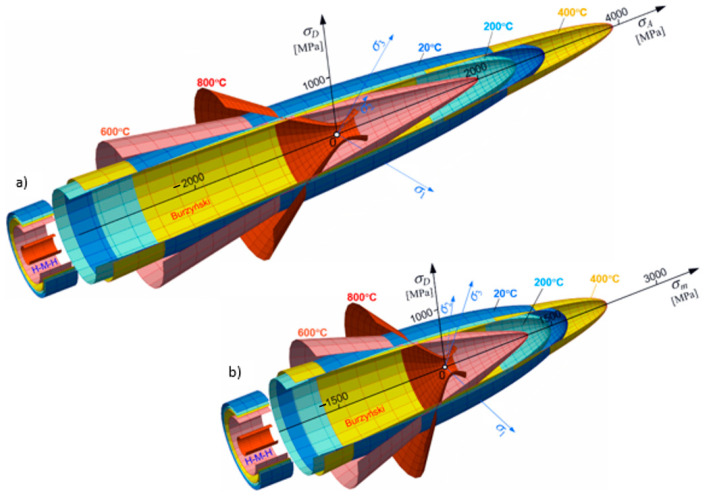
Full temperature-dependent evolution of limit surface for steel St12T: (**a**) the set of surfaces are described in the function of axiatoric stress; (**b**) the set of surfaces are depictured in the function of averaged normal stresses.

**Figure 15:**
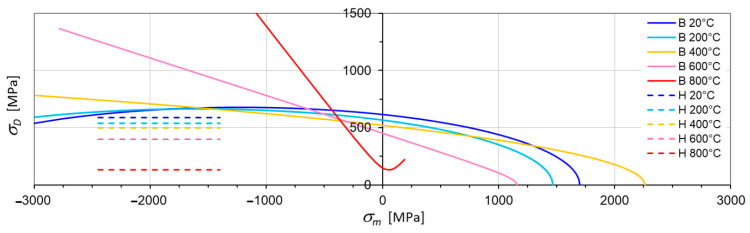
Cross-sections of the yield surface intersected by the hydrostatic plane in St12T steel according to the HMH hypothesis (H) and the Burzyński hypothesis (B).

The yield surfaces given by Equation (30) are presented in Figure 14. The presented yield surfaces take into account changes driven by temperature in the cycle asymmetry coefficient and the offset values of tensile yield strength. Those surfaces were established for temperatures given in Table 2. The points at which plastic regions are intersected by the hydrostatic plane, i.e., a plane defined by axis $\sigma_{D}$ (Figure 14) in the deviatoric plane, and the hydrostatic axis $\sigma_{1}=\sigma_{2}=\sigma_{3}$ (perpendicular to the deviatoric plane), are presented in Figure 15, plotted by measuring average normal stress $\sigma_{m}$ on the hydrostatic axis and deviator stress $\sigma_{D}$ on the deviator stress axis (projection of the sum of vectors of the main stress components in plastic regions on the deviatoric plane—$\sigma_{D}={\left|\vec{\sigma_{1}}+\vec{\sigma_{2}}+\vec{\sigma_{3}}\right|}_{D}=\sqrt{2}\sigma_{m}$) [21]. The deviatoric stress, in general, describes a circumferential change in the yield stress deviatoric section; it describes “the waves of third invariant” but, in the case under consideration, is circumferentially constant and equal to $\sigma_{HMH}$.

Now, let us analyze the cases of limit surfaces which also take into account temperature dependence of $\tilde{\nu }(T)$ (see: Figure 13). In Figure 14, the full temperature dependence of the limit surface is shown; the word “full” means that the evolution is fully three parametrical: κ(T), $\tilde{\nu }(T)$, and $k_{t}(T)$. In Figure 14a, the set of surfaces is described in the function of axiatoric stress; however, in Figure 14b, the same set of surfaces is depictured in the function of averaged normal stresses for steel St12T.

An analysis of the shape of the yield surfaces in Figure 14 and their cross-sections in Figure 15 indicates that the Burzynski’s hypothesis illustrates the phenomenon of blue brittleness to a better extent than the HMH hypothesis, and the surface obtained for 400 °C falls within the scope of the phenomenon. The blue brittleness is caused by the deformation aging of carbon and nitrogen interstitial atoms in the temperature range of 200 to 450 °C [53]. The asymmetry coefficient ϰ appears to exert a critical influence at a temperature of around 400 °C, where a local minimum is observed (Figure 14). As a result, the plastic region occurs even at temperatures lower than 200 °C (Figure 15).

**Figure 16:**
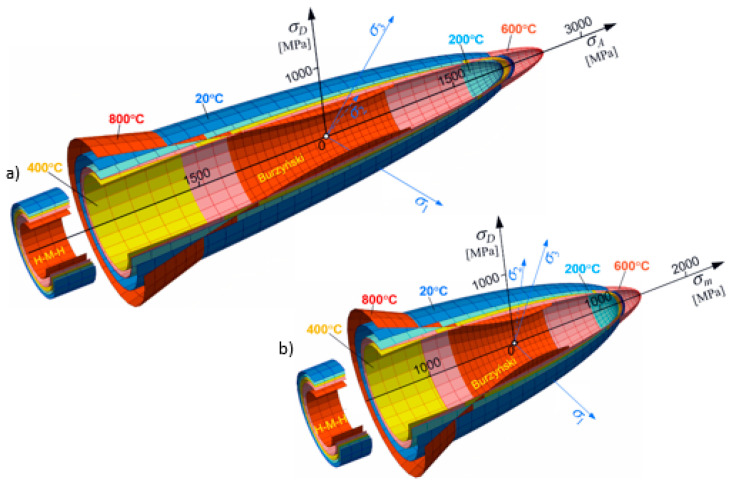
Full temperature-dependent evolution of limit surface for steel 26H2MF: (**a**) the set of surfaces are described in the function of axiatoric stress; (**b**) the set of surfaces are depictured in the function of averaged normal stresses.

The second steel, 26H2MF, has another property. In Figure 16a, the set of surfaces is described in the function of axiatoric stress; however, in Figure 16b, the same set of surfaces is depictured in the function of averaged normal stresses.

## 5. Thermo-Mechanical Analysis of a Turbine Blade

### 5.1. Specifics of the High Thermal Loading

A simple numerical example, with some similarities to real conditions that occur during a very short thermal cycle, was conducted in the following section. Such cycles are of great importance due to the impact of wind and solar power station in the classical one. The following analysis was restricted only to a thermal loading caused by hot and cold working fluid; the curve of changing the steam temperature is presented in Figure 22 and, additionally, in the role of a “time-like” co-ordinate in Figure 25. The steam pressure drop was omitted in this analysis, since we were interested only in pure thermal effort.

### 5.2. Formulation of Coupled Problem

The aim of the present section is recognition of the difference level between the Huber–Mises–Hencky (HMH) and the Burzyński approaches with regard to yield surface, equivalent stress, and temperature-dependent elasto-plastic behavior. As an example, a proportionally rescaled turbine blade was simulated using the cycling-plasticity Chaboche model, equipped additionally with the Burzyński three-parametrical yield surface (Equation (30)). For discretization of a set of governing equations, the finite element method was used; the aim of analysis was to simulate the behavior of a blade in real working conditions to give some comparisons in stress–strain behavior described by the well-known model HMH (with strength symmetry) with proposed Burzyński hypothesis (with strength differential effect). Let us underline, once again, the state of stresses and strains were analyzed by using the validated Chaboche model, equipped with temperature-dependent coefficients. The process of heating and cooling a turbine blade was realized by coupled CFD analysis at fluid and CSD analysis in the solid of the blade. This procedure was described and tested in a few works of the authors [18,36].

The stress tensor σ is obtained on the basis of the generalized Hooke law, as follows:

(39) $$\sigma =C(\varepsilon -\varepsilon_{pl}-\varepsilon_{th})$$

where ε is the total tensor and $\varepsilon_{pl}$ is the plastic strain tensor; C denotes the fourth-order isotropic elastic tensor, defined by the temperature-dependent elastic modulus E(T) and by the Poisson ratio ν(T). Additionally, $\varepsilon_{th}=\alpha (T)\Delta TI$ is a spherical thermal strain tensor. The model assumes associated plastic flow:

(40) $${\dot{\varepsilon }}_{pl}={\dot{\bar{\varepsilon }}}_{pl}\frac{\partial f}{\partial \sigma }$$

where ${\dot{\varepsilon }}_{pl}$ represents the plastic strain rate tensor and ${\dot{\bar{\varepsilon }}}_{pl}$ is the von Mises equivalent plastic strain rate, defined as ${\dot{\bar{\varepsilon }}}_{pl}={\left(\frac{2}{3}{\dot{\varepsilon }}_{pl.ij}{\dot{\varepsilon }}_{pl.ij}\right)}^{1/2}$. The pressure sensitive Burzyński yield function (Equation (23)), allowing for isotropic and kinematic hardening, is defined as:

(41) $$f=\frac{2}{3}\left(1+\tilde{\nu }\right){\overset{ˇ}{\sigma }}_{HMH}^{2}+3\left(1-2\tilde{\nu }\right)p^{2}+3(\varkappa -1){\overset{ˇ}{k}}_{t}\;p-{\varkappa \overset{ˇ}{k}}_{t}^{2}=0$$

where ${\overset{ˇ}{\sigma }}_{HMH}^{2}=3{\overset{ˇ}{J}}_{2}=\frac{3}{2}(s_{ij}-\alpha_{ij})(s_{ij}-\alpha_{ij})$ denotes the equivalent HMH stress for kinematic hardening with respect to the back stress $\alpha =\sum_{m=1}^{3}\alpha_{m}$ and ${\overset{ˇ}{k}}_{t}=k_{t}+R$ denotes tension yield with isotropic hardening described by Chaboche strengthening R as:

(42) $${\dot{\alpha }}_{m}=\frac{2}{3}C_{m}{\dot{\varepsilon }}_{pl}-\gamma_{m}\alpha_{m}{\dot{\bar{\varepsilon }}}_{pl}+\frac{1}{C_{m}}\frac{\partial C_{m}}{\partial T}\alpha_{m}\dot{T};\;\;\;\dot{R}=b(Q-R){\dot{\bar{\varepsilon }}}_{pl}$$

where $C_{m},\;\gamma_{m},\;b,\;Q$; m=1,2,3 are constants to be calibrated for a specific steel. In the first step, the classical Chaboche cyclic plasticity model [54] has been extended to be a temperature-dependent model. The extension has been performed by introducing temperature-dependent model coefficients, density—ρ(T); Young modulus—E(T); Poisson ratio—ν(T); yield stress in tension—$k_{t}(T)$; strength differential ratio—ϰ(T); specific heat capacity—$c_{p}(T)$; linear coefficient of thermal expansion—α(T); coefficient of thermal conductivity—λ(T); and Chaboche coefficients $C_{1}(T)$ and $\gamma_{1}(T)$, into the commercial code. All of these temperature dependence model coefficients were measured by authors in separate papers (see: Refs. [19,20,21]). The next step was to add the Burzyński surface. The UMAT procedure was prepared separately by Kamil Banaś during his PhD thesis and published in a paper [55].

### 5.3. Thermal–Mechanical Validation Test

A thermo-mechanical compression test of the specimens was performed for subsequent validation of the model [21]. Then, an attempt was made to numerically simulate the experiment involving thermo-mechanical loading of a cylindrical specimen in the elastic–plastic range. The results of the numerical analysis were analyzed and compared with the data obtained from the experiment. The stresses of the specimen, characterized by stress intensities according to the HMH and Burzyński hypotheses, were compared with the normal compressive stresses of the specimen, which were determined from experimental data [21].

**Figure 17:**
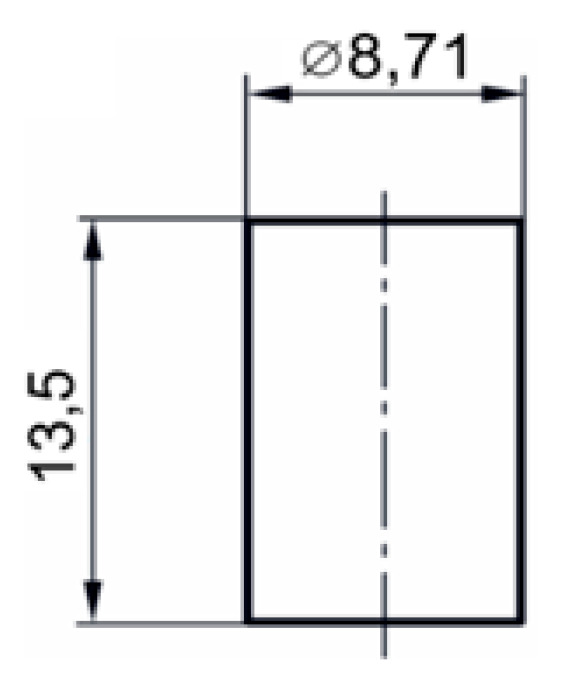
Dimensions of the sample used for the thermo-mechanical compression test [21].

A cylindrical specimen with the initial dimensions shown in Figure 17 was used in the experiment discussed in the present paragraph.

**Figure 18:**
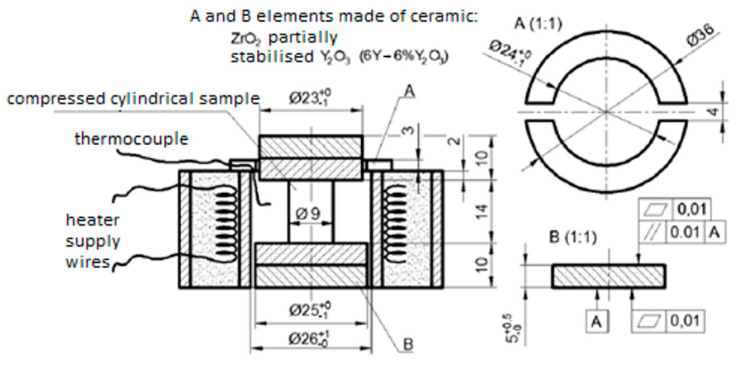
Scheme of the stand used for the thermo-mechanical compression test [21].

The St12T steel specimen was placed in the testing machine inside a specially designed heating chamber capable of heating the specimen to 450 °C (Figure 18). During the test, the value of the force and the corresponding displacement between the extreme B plates was measured (Figure 18). The displacement was corrected for the deformability of the B plates made of ZrO_2_ ceramics. The low thermal conductivity of the order of 2 W·m^−1^·K^−1^ of this ceramic prevented heat transfer from the sample to the machine grips, and the low coefficient of friction partially compensated for the effect of friction on the contact surfaces. In addition, mechanical properties like Young’s modulus of 270 GPa, hardness exceeding 71HRC, compressive strength of more than 2100 MPa, and coefficient of thermal expansion 1 × 10^−5^ K^−1^ remain almost unchanged up to temperatures above 500 °C [21].

**Figure 19:**
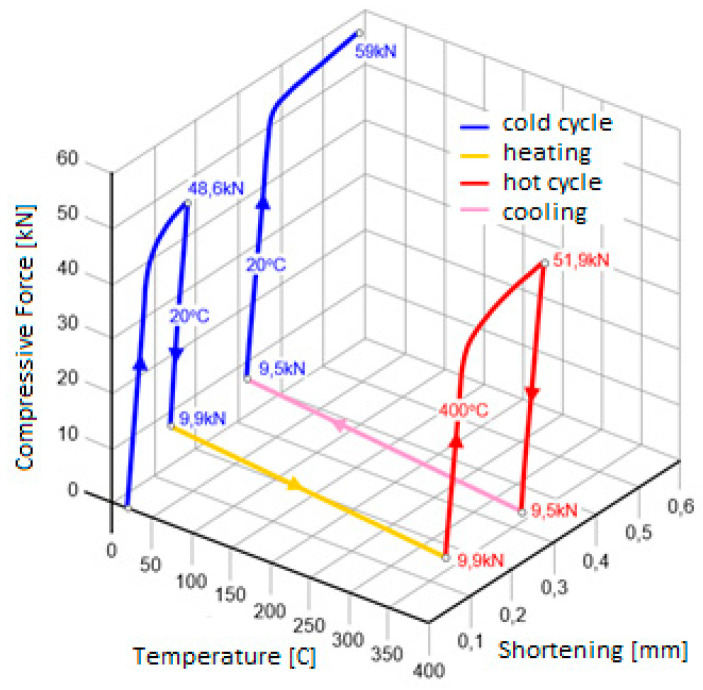
Cycle of thermomechanical compression of the sample [21].

The thermo-mechanical loading cycle of the sample is shown in Figure 19. It began by compressing the sample at ambient temperature (20 °C) to a force of 48.6 kN. Then, the specimen was unloaded to a force of 9.9 kN and the heating of the specimen to 400 °C was started, maintaining the same value of the compressive force. Then, at constant temperature, the specimen was again loaded to 51.9 kN and relieved to 9.5 kN. After that, the cooling of the sample was started while maintaining a constant value of the compressive force. In the final step, when the sample reached the temperature of 20 °C, the sample was again loaded with a compressive force to 59kN and relieved [21].

A numerical analysis was carried out on the three-dimensional geometry of the specimen discretized with a structural mesh, with 20-node second-order finite elements of Hex-20 type. The same thermo-mechanical loading cycle was programmed as in the experiment (Figure 19).

**Figure 20:**
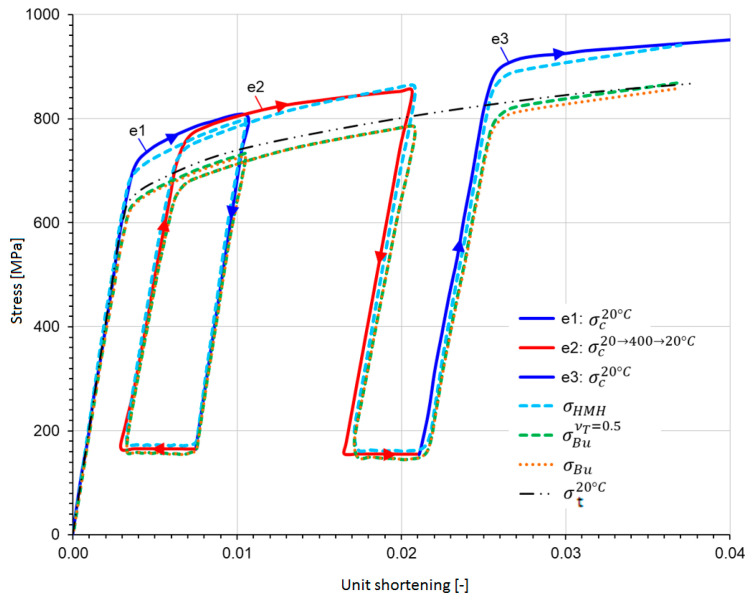
The compressive stress $\sigma_{C}$ determined from experiment (temperature stages: e1, e2, and e3 of a single thermomechanical cycle) and the corresponding equivalent stresses $\sigma_{HMH}$ and $\sigma_{Bu}$ from the numerical simulation; in addition, a stress plot from the tensile test (e) [21].

Figure 20 shows, determined from the experiment, the compressive stress as a function of unit shortening, which was divided into three stages: two so-called cold stages (20 °C) marked e1 and e3 and one hot stage e2, in which the temperature increased from 20 °C to 400 °C and, after mechanical relief, decreased again to 20 °C. After numerical simulation, equivalent stresses were determined according to the Huber–Mises–Hencky hypothesis ($\sigma_{HMH}$) and the Burzyński hypothesis ($\sigma_{Bu}$), the courses of which are shown in Figure 20 [21].

A comparison of the graphs in Figure 20 shows that, for the example considered here, the inclusion of the plasticity factor $\nu_{T}$ has slightly corrected the values of Burzynski’s equivalent stress, which were determined assuming $\nu_{T}$ = 0.5.

Seemingly, it would appear that the HMH hypothesis better reproduces the experimental results. However, it should be remembered that $\sigma_{HMH}$ was obtained from calibrating the model on compression rather than tension curves. And, for both hypotheses, the equivalent state is that of uniaxial tension. So Burzynski’s hypothesis here only relates the compression state to the tension state, which is the idea behind it by means of the $\varkappa_{T}$ factor in particular. For comparison, the tensile curve ($\sigma_{t}^{20\;{}^{\circ}C}$) of one of the samples from the aforementioned study is also included in Figure 20. Of course, the $\sigma_{HMH}$ stresses obtained from the compression curves calibrated on the graphs can be related, without making a major mistake here, to the stress obtained from the experiment, since a practically uniaxial state of stress occurs here. On the other hand, it should be remembered that the complex stress state described by the two hypotheses considered here is equivalent to the uniaxial tensile state. If one were to calibrate the model parameters on the tensile plots, the $\sigma_{HMH}$ stresses would no longer reflect the experiment, since plasticization would occur much earlier than in compression.

### 5.4. Thermal Loading and Boundary Data

**Figure 21:**
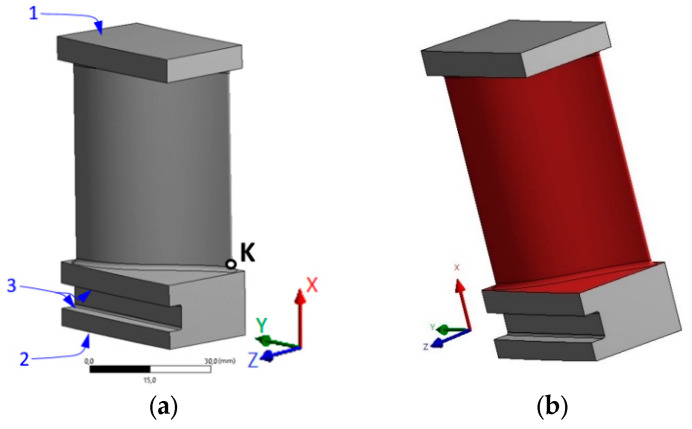
Geometry of the analyzed turbine blade: (**a**) constraints imposed on the surfaces; (**b**) heated or cooled surfaces (red). These specified boundary conditions represent the real operating process of the blade.

**Figure 22:**
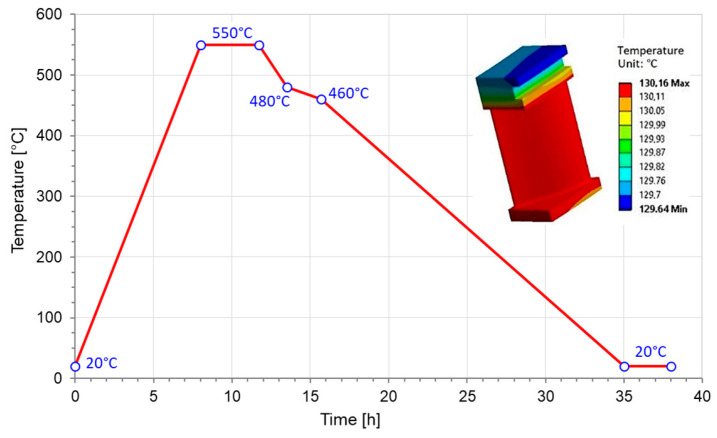
Changes in the inlet temperature during blade heating and cooling cycles and the temperature field within the blade after 1 h.

The crucial point is the boundary condition of a blade. It was mounted without slack in direction x between nondeformable walls, denoted by blue lines 1, 2, and 3 in Figure 21a. The surface of the blade, as well as the adjacent surfaces of the blade shoulder and the flange were heated and cooled by working fluid (Figure 21b). Good agreement with results and conclusions was noted following from more fundamental heat exchange models (see: Refs. [17,35]). Changes in temperature of working fluid over the one cycle time are presented in Figure 22.

A sequentially coupled procedure was used for the thermo-mechanical simulation in the Ansys 17.1 software. The analysis was carried out on a scaled-down 400 MW high-pressure turbine blade model shown in Figure 21, with fabricated heat-resistant steel St12T. For the heat transfer analysis, a 10-node quadratic heat transfer tetrahedron element type was used, while, for the thermo-mechanical analysis, a 10-node quadratic tetrahedron element type was used [56]. A text element shape with a global seed size of 0.8 mm was used for the analysis. The temperature distribution profile is nonstationary and leads to significant thermal stresses.

### 5.5. Results of Numerical Simulations

Two series of calculations were performed—one based on the HMH hypothesis and the second on the Burzyński hypothesis. In both cases, model coefficients were dependent on temperature. The main effect is observed due to temperature-driven changes in the strength differential asymmetry coefficient given in Figure 12.

**Figure 23:**
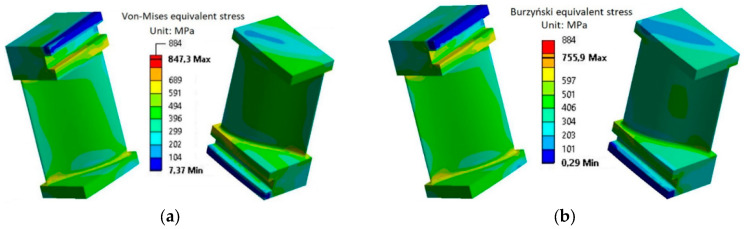
The stress–strain behavior of the analyzed turbine blade according to (**a**) the HMH hypothesis and (**b**) the modified Burzyński hypothesis after blade heating to 550 °C.

During analysis, the full state of thermally driven displacement, stresses, and elastic, thermal, and plastic strains is obtained. For instance, the equivalent stresses of the analyzed blade at a temperature of 550 °C described by the HMH hypothesis (Figure 23a) and the Burzyński hypothesis (Figure 23b) are presented in Figure 23. Both $\sigma_{HMH}$ and $\sigma_{Bu}$ equivalent stresses are related via Equation (31).

**Figure 24:**
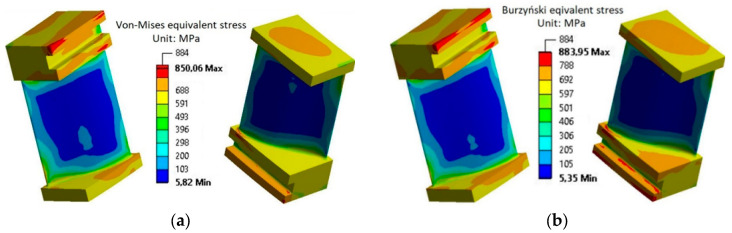
The stress–strain behavior of the analyzed turbine blade according to (**a**) the HMH hypothesis and (**b**) the modified Burzyński hypothesis after blade cooling to 20 °C.

Similarly, the state of equivalent stresses–strain of the analyzed turbine blade cooled to 20 °C described by the HMH hypothesis and the Burzyński hypothesis is presented in Figure 24a,b.

Relatively high equivalent stress is observed in the vicinity of point K; it follows that the possible location of crack is a place denoted by point K in Figure 21a. These results also suggest that blades had been tightly packed inside the turbine casing during assembly. As a result, shoulder grooves can be stuck (heat-damaged) inside the casing during turbine operation. Therefore, numerical simulations of the heating–cooling cycle (Figure 22) were performed in view of the constraints imposed on surfaces 1, 2, and 3 in Figure 21a. It should be noted that the described variant of the thermo-mechanical loading cycle with the indicated constraints was only one of the many numerically simulated variants.

**Figure 25:**
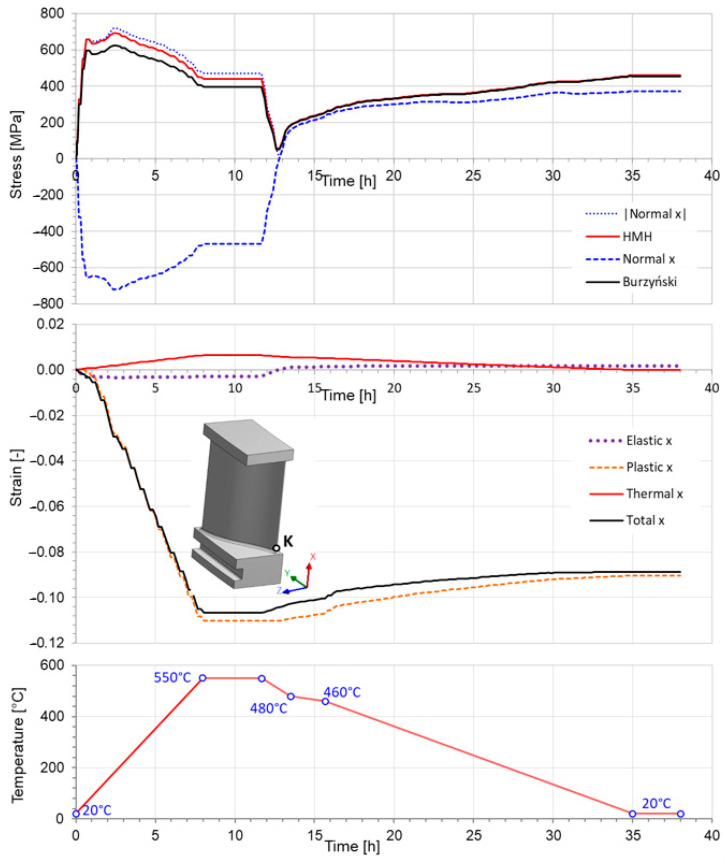
(**top**) Changes in $\sigma_{xx}$, HMH equivalent stress and Burzyński equivalent stress, and (**middle**) in strain at point K during the thermomechanical loading cycle (**bottom**).

Changes over time (and, consequently, temperature-dependent changes) in the values of the HMH equivalent stress and Burzyński equivalent stress, changes in normal stress in the direction of the x axis, total deformation in the direction the x axis, as well as changes in the main components of the stress tensor (elastic, plastic, and thermal deformation) at point K (Figure 21) are presented in Figure 25. To compare the changes in stress values, the normal component of the stress tensor $\sigma_{xx}$ at point K was reflected across the time axis (Figure 25). For obvious reasons, only the negative values of the normal component of the stress tensor were reflected. At point K, the values of $\sigma_{xx}$ clearly dominated over the remaining components of the stress tensor, which was confirmed by a comparison of the remaining results of the numerical analysis.

An analysis of stress and strain values (Figure 25) indicates that, under the adopted simulation conditions, plastic strain began to evolve already in the initial heating phase when temperature was considerably below 550 °C.

Finite element analysis of a turbine blade under thermo-mechanical conditions revealed that the value of the Burzyński equivalent stress is lower than the value of the HMH equivalent stress at point K when compressive force is prevailing in a region near point K (Figure 25). Moreover, both energetic hypotheses provide comparable values of the equivalent stress when tensile stresses occur. It does not mean that HMH will always be higher or equal to the Burzyński equivalent stress. For example, the analysis of the turbine blade under high-temperature working conditions can show that the values of the Burzyński equivalent stress exceed the values of the HMH equivalent stress in some cases.

The top part of Figure 25 gives a good comparison of the values of the HMH equivalent stress and the Burzyński equivalent stress. In the peak of the compression of the blade due to heating up to temperature of 550 °C, the value of the HMH equivalent stress exceeds the value of the Burzyński equivalent by approximately 90 MPa. Tensile forces occur in the turbine blade during the stage of cooling to a temperature of 20 °C. In this case, the value of the Burzyński equivalent stress exceeds the value of the HMH equivalent stress by 34 MPa in the last stage of the cooling process.

## 6. Summary and Conclusions

It is shown that the concept of material effort described in terms of three basic features, the stress intensity, the stress triaxiality, and the stress shearness, leads to the three-parameter limit surface that is a simple extension of three-parameter Burzyński thermo-mechanical effort. It appears that the proposed model can be simply calibrated by three experiments: one-axial tension, one-axial compression, and pure shear tests. The model was tested experimentally for heat-resistant St12T and 26H3t steel. Thereafter, it was used to describe a behavior of a turbine blade under thermo-mechanical operating conditions. The loading by the thermal cycle was designed so that the heat-resistant steel undergoes a wider range of yield strength values that change disproportionally relative to changes in temperature. Significant differences appear between the HMH and Burzyński approaches caused by appearing compression and tension during the thermal cycle.

Indeed, the initiation and evolution of the plasticity region in St12T steel indicates that temperature exerts a strong influence on yield strength, in particular, in the region of tension, i.e., in the region where ${(\sigma }_{1}+\sigma_{2}+\sigma_{3})>0$. At temperatures higher than 800 °C, the yield surface behavior of the analyzed steel tends to be zero in the region of tension [30].

Let us note that differences between both approaches exist not only in redistributions of elastic stresses but also in the plastic domain, where the rate of plastic strain, in one case, is governed by $\sigma_{HMH}$ and, in the other case, by $\sigma_{Bu}$. Details of this influence have been researched and discussed in [36]; however, generally, due to another (noncylindrical) shape of the Burzyński yield surface, this influence is more important within the tension domain [30].

This paper has shown the importance of the influence of temperature on the behavior of the yield surface and internal forces in steels under consideration. It is worth mentioning that the Burzyński hypothesis can illustrate the blue brittleness phenomenon in a better way than the HMH hypothesis.

## Figures and Tables

**Table 1:** Chemical composition of analyzed steels [21].

Steel		Chemical Composition (%)							
Grade	Signature	C	Si	V	Cr	Mn	Ni	Cu	Mo
26H2MF	24CrMoV55	0.23	0.52	0.25	1.54	0.30	0.12	0.17	0.60
St12T	X22CrMoV12-1	0.16	0.37	0.24	11.10	0.44	0.42	0.13	0.96

**Table 2:** Plasticity coefficient $\nu_{T}$ and coefficient of asymmetry $\varkappa_{T}$ at elevated temperatures.

Steel	Temperature	Yield Stress (MPa)			Coefficient (–)	
	(°C)	$R_{0.2r}^{T}$	$R_{0.2c}^{T}$	$R_{0.3s}^{T}$	$\varkappa_{T}$	$\nu_{T}$
St12T	20	720.3	786.0	436.37	1.091	0.4866
	200	656.7	731.7	401.76	1.114	0.4883
	400	608.0	666.3	367.51	1.108	0.4997
	600	487.0	639.3	319.28	1.313	0.5271
	800	160.0	290.0	197.65	1.813	1.1033
26H2MF	20	667.3	779.0	417.60	1.167	0.4905
	200	610.7	693.3	377.50	1.135	0.4855
	400	567.0	628.0	346.06	1.108	0.4866
	550	507.3	573.3	311.37	1.130	0.5001
	700	388.5	460.0	238.60	1.184	0.5696

## Data Availability

Data will be made available on request.
